# Extent of routine diagnostic cardiac work-up at certified German stroke units participating in the prospective MonDAFIS study

**DOI:** 10.1186/s42466-023-00246-8

**Published:** 2023-06-01

**Authors:** Manuel C. Olma, Serdar Tütüncü, Ulrike Grittner, Claudia Kunze, Muhammad Jawad-Ul-Qamar, Paulus Kirchhof, Joachim Röther, Götz Thomalla, Roland Veltkamp, Ulrich Laufs, Darius G. Nabavi, Peter U. Heuschmann, Matthias Endres, Karl Georg Haeusler

**Affiliations:** 1 Center for Stroke Research Berlin, Charité - Universitätsmedizin Berlin, Berlin, Germany; 2 Department of Neurology with Experimental Neurology, Charité - Universitätsmedizin Berlin, Berlin, Germany; 3 Department of Internal Medicine, Herz-Jesu- Hospital Dernbach, Dernbach, Germany; 4 Institute for Biometry Und Clinical Epidemiology, Charité - Universitätsmedizin Berlin, Berlin, Germany; 5 Institute of Cardiovascular Sciences, College of Medical and Dental Sciences, Medical School, University of Birmingham, Birmingham, UK; 6 University Heart and Vascular Center Hamburg, Hamburg, Germany; 7 German Center for Cardiovascular Research, Partner Site Hamburg/Kiel/Lübeck, Hamburg, Germany; 8 Department of Neurology, Asklepios Hospital Altona, Hamburg, Germany; 9 Department of Neurology, University Medical Center Hamburg-Eppendorf, Hamburg, Germany; 10 Department of Neurology, Alfried Krupp Hospital, Essen, Germany; 11 Department of Brain Sciences, Imperial College London, London, UK; 12 Department of Cardiology, University Hospital, Leipzig University, Leipzig, Germany; 13 Department of Neurology, Vivantes Hospital Neukölln, Berlin, Germany; 14 Institute of Clinical Epidemiology and Biometry, University Würzburg, Würzburg, Germany; 15 Clinical Trial Center Würzburg, Institute of medical Data Science, University Hospital Würzburg, Würzburg, Germany; 16 Berlin Institute of Health (BIH), Berlin, Germany; 17 German Center for Neurodegenerative Diseases (DZNE), Partner Site Berlin, Berlin, Germany; 18 German Center for Cardiovascular Research (DZHK), Partner Site Berlin, Berlin, Germany; 19 German Center for Mental Health (DZPG), Partner Site Berlin, Berlin, Germany; 20 Department of Neurology, University Hospital Würzburg, Josef-Schneider-Str. 11, 97080 Würzburg, Germany; 21 Castle Hill Hospital, Hull University Teaching Hospitals, Cottingham, UK; 22 Department of Neurology, Alexianer St. Josefs-Krankenhaus Potsdam, Potsdam, Germany

**Keywords:** Ischaemic stroke, Transient ischaemic attack, atrial fibrillation, Stroke-unit monitoring, echocardiography, Holter-ECG

## Abstract

**Background:**

About 25% of all ischaemic strokes are related to cardio-embolism, most often due to atrial fibrillation (AF). Little is known about the extent and standardization of routine cardiac diagnostic work-up at certified stroke-units in Germany.

**Methods:**

The MonDAFIS study included non-AF patients with acute ischaemic stroke or transient ischaemic attack (TIA) at 38 certified stroke-units in Germany. Here, we analysed routine diagnostic work-up and disregarded study-related Holter-ECG monitoring. We compared duration of stroke-unit stay, number of 24-h Holter-ECGs, and echocardiography performed between university-based comprehensive stroke centres (UCSC, 12 hospitals, 1606 patients), non university-based comprehensive stroke centres (nUCSC, 14 hospitals, 892 patients), and primary stroke centres at non-university hospitals (PCS, 12 hospitals, 933 patients) using multivariable mixed regression analyses. Detection of a first AF episode in-hospital was also compared between hospitals of different stroke-unit levels.

**Results:**

In 3431 study patients (mean age 66.2 years, 39.5% female, median NIHSS = 2 on admission), median duration of the stroke-unit stay was 72 h (IQR 42–86). Stroke-unit stay was longer (categorised ≤ 24/ > 24- ≤ 72/ > 72 h) for patients with severe stroke (NIHSS score ≥ 5/ < 5: OR = 1.6, 95%CI 1.3–2.0) and for patients with ischaemic stroke vs. TIA (OR = 1.7, 95%CI 1.4–2.1). Overall, 2149/3396 (63.3%) patients underwent at least one additional 24-h Holter-ECG (median 1 [IQR 0–1], range 0–7). Holter-ECG rate was 47% in UCSC, 71% in nUCSC, and 84% in PCS. Compared to PCS, AF was less often detected in-hospital in UCSC (OR = 0.65, 95%CI 0.45–0.93) and nUCSC (OR = 0.69, 95%CI 0.46–1.04). Transoesophageal echocardiography (TEE) only was performed in 513/3391 (15.1%) study patients, transthoracic echocardiography (TTE) only in 1228/3391 (36.2%), and TEE combined with TTE in 1020/3391 (30.1%) patients. Patients younger than 60 years (vs. ≥ 60 years) underwent TEE more often than those older than 60 years (OR = 3.44, 95%CI 2.67–4.42). TEE (IQR 34–65%) and TTE rate (IQR 40–85%) varied substantially among study centres. Echocardiography rate (TTE and/or TEE) was 74.0% in UCSC, 85.4% in nUCSC, and 90.3% in PSC, respectively.

**Conclusions:**

In the MonDAFIS study, the routine use of echocardiography and Holter-ECG monitoring varied in participating stroke centres and at stroke-unit level, if grouped according to stroke-unit certification grade and hospitals´ university status.

*Trial registration* Clinical Trials, NCT02204267. Registered 30 July 2014, https://clinicaltrials.gov/ct2/show/NCT02204267.

**Supplementary Information:**

The online version contains supplementary material available at 10.1186/s42466-023-00246-8.

## Introduction

Patients with acute stroke or transient ischaemic attack (TIA) benefit from stroke unit treatment, which is associated with increased survival, a higher rate of functional independence and with a greater likelihood of living at home one year after stroke [8, 9]. In Germany, acute stroke care is provided by more than 340 certified stroke units at present and well-organized following distinct certification criteria [10, 11]. Approximately 10% of all certified stroke units in Germany are university-based comprehensive stroke centres (UCSC), 40% are non university-based comprehensive stroke centres (nUCSC) and 50% are primary stroke centres (PSC). To be certified as comprehensive stroke centre, at least 500 stroke patients per year have to treated using at least six stroke unit beds and endovascular treatment availability 24/7 [10, 11].

In addition to acute stroke treatment and the prevention of stroke-associated complications, one main focus of stroke unit treatment is clarification of stroke aetiology. In line with this, the European Stroke Action Plan of the European Stroke Organisation (ESO) states that access to key investigational modalities is one of the main targets for stroke care in 2030 [12]. The importance of ECG diagnostic, such as resting ECG, 24-h Holter-ECG and rhythm monitoring in stroke units, as well as echocardiography are high-lighted by the fact that approximately 25% of all ischaemic strokes are caused by cardio-embolism, most often due to atrial fibrillation (AF). AF is first detected in about 5% of all ischaemic stroke patients by in-hospital ECG monitoring [4], most often resulting in oral anticoagulation in secondary stroke prevention [7, 16]. Moreover, heart failure, patent foramen ovale or endocarditis may lead to ischaemic stroke or TIA, but can be detected by echocardiography—the gold standard of cardiac imaging in stroke patients [15].

As there is no randomized controlled trial demonstrating stroke prevention or reduced mortality by prolonged ECG monitoring or echocardiography after ischaemic stroke, respective guideline recommendations are rather vague, emphasizing patient selection as paramount, as diagnostic capacities are limited to some extent even in industrialized countries. Despite the fact that cardiac work-up is relevant for the vast majority of stroke patients to exclude a cardiac source of stroke, strict selection criteria for patients who should undergo cardiac work-up are not established in clinical practice, as guideline recommendations are vague. Subsequently, cardiac work-up varies among stroke centres [3, 14], but respective data from prospective randomized trials are sparse. In Germany, physicians who treat stroke patients at certified stroke units are strongly encouraged to provide cardiologic diagnostic workup to fulfil predefined quality criteria of the certification process [10, 11]. Interestingly, little information is available whether routine cardiac diagnostic work-up also differs at stroke unit level. Of note, physicians who treat stroke patients at certified German stroke units are strongly encouraged to provide cardiologic diagnostic workup to fulfil the predefined quality criteria of the certification process [10, 11].

The aim of the present analysis is to characterize the extent of routine cardiologic examinations during routine work-up, in particular the extent of routine ECG monitoring and echocardiography procedures. We therefore analysed data of the investigator-initiated prospective MonDAFIS study, which randomised 3465 patients with acute ischaemic stroke or TIA but without known AF to usual diagnostic care or usual diagnostic care plus additive systematic Holter-ECG monitoring for up to seven days in-hospital. In the intervention group, core-lab based ECG analysis increased AF detection rate in-hospital, but neither impacts on oral anticoagulation rate after 12 months or on vascular events or death within 24 months after the index stroke, as published previously [6]. As stroke unit monitoring duration, use of Holter-ECGs or echocardiography was left at the discretion of the treating physicians, we are able to report the extent of routine cardiac work-up after the index-stroke/TIA. Grouping the 38 participating stroke centres according to stroke unit level, we analysed the impact of routine cardiac diagnostic work-up on AF detection in-hospital in addition.

## Methods

### Study design

The *Impact of standardized MONitoring for Detection of Atrial Fibrillation in Ischaemic Stroke* (MonDAFIS) study (ClinicalTrials.gov, NCT02204267) was an investigator-initiated, prospective multicentre study, sponsored by the Charité - Universitätsmedizin Berlin, Germany and supported by an unrestricted research grant from Bayer Vital GmbH, Germany to the Charité. The Bayer Vital GmbH, Germany had no influence on study design, data analysis, and publication. The study was designed to evaluate the additive value of systematic in-hospital ECG monitoring (using Holter-ECG for up to seven days on top of usual diagnostic care) after acute ischemic stroke or transient ischemic attack with respect to the rate of patients treated with oral anticoagulation 12 months after the index stroke (primary endpoint). Main results were published previously, along with the study protocol and the statistical analysis plan [5, 6]. The aim of the present analysis is to characterize the extent of routine cardiologic examinations during routine work-up, in particular the extent of routine ECG monitoring and echocardiography procedures. Because there are different levels of stroke unit certification established in Germany [10], an analysis of possible effects of stroke unit level on routine cardiac work-up as well as an approximately equal distribution of study sites according to stroke unit level was planned as part of the study protocol.

### Study population and study intervention

Patients were eligible for study enrolment if they were ≥ 18 years of age, had an index stroke defined as ischaemic stroke or a TIA (with documented neurological deficit at hospital admission by a neurologist or an acute ischaemic lesion on brain MRI). Study patients had to be admitted to a certified stroke unit in Germany within 72 h after stroke onset. Patients were excluded if AF was known before randomisation (for details, see [6]).

Study patients were randomised 1:1 to continuous Holter-ECG recording for up to 7 days in-hospital stay on top of standard diagnostic care (intervention group) or standard diagnostic care (control group). Of note, the results of the study-ECG were not included in the present analysis, focussing on routine diagnostic cardiac work-up. Study-specific diagnostic recommendations included a baseline 12-channel ECG on hospital admission and at least 24 h of (monitor-based) ECG monitoring on the stroke unit. Additional ECG monitoring and echocardiography was performed at the discretion of the treating physicians, independent of randomisation [6].

### Statistical analysis

The complete randomised dataset was used for this secondary data analysis including all 3431 patients randomised to the standard of care or intervention group fulfilling major inclusion and exclusion criteria and have no lack of any data post randomisation. We used Fisher’s exact test, one-way analysis of variance for independent samples or Kruskal–Wallis-test to compare stroke-unit levels regarding differences in selected independent variables. In order to test the association of (a) type of echocardiography (transthoracic/transoesophageal); (b) number of 24-h Holter-ECG (0, 1, ≥ 2); (c) duration of the stroke unit stay, (d) loop recorder implantation in hospital, and e) AF detection in hospital, we used multiple mixed regression models with appropriate link functions according to the scaling of the outcome with random intercepts for the study centres. Regarding a) and b) a mixed multivariable multinomial logistic regression analyses was used including sex (female/male), age (< 60 years/ ≥ 60 years), stroke severity (National Institutes of Health Scale (NIHSS) score < 5/ ≥ 5 points), index stroke or TIA and stroke unit level (UCSC, nUCSC or PCS) as independent variables, and adjusting for randomisation group (intervention vs. control group) and total duration of hospital stay (days). For the following two multinomial logistic regression analyses, the diagnostic procedures (i.e., independent variable of interest) were compared to no diagnostic procedures (i.e., reference category of the independent variable of interest).

Regarding (c) we used a mixed ordinal logistic regression model categorising the independent variable (duration of the stroke unit stay) in three groups, i.e., ≤ 24 h, > 24–72 h, > 72 h, controlling for the identical covariates as in the multinomial logistic egressions except for age (< 60/60–74/ ≥ 75 years). Regarding (d) and (e) we performed mixed binary logistic regression analyses, using the same covariates as in (c) with the exception that in (e) “total duration of hospital stay” was not included in the model. An additional subgroup analyses was conducted in analogy to e) in which the dependent variable was “AF detection during stroke unit monitoring”. All data were processed using SPSS 28.0 for Windows (SPSS, Chicago, III., USA). A two-sided significance level of α = 0.05 was used. No multiplicity adjustments were done in this secondary data analysis. Therefore, the results have to be considered exploratory, and p-values should be interpreted cautiously. Interpretation of results is mainly based on effect measures and 95% confidence intervals.

## Results

### Study cohort

Mean age of 3431 study patients was 66.2 years (± 12.9), 39.5% were female and 30.1% had a TIA as qualifying event. The median NIHSS score on hospital admission was 2 points (IQR 1-4), as published previously [6]. Overall, 1606 study patients (46.8%) were admitted to one of 12 UCSC, 892 study patients (26.0%) were admitted to one of 14 nUCSC, and 933 study patients (27.2%) were admitted to one of 12 PSC. Baseline characteristics of study patients differed at stroke unit level. PCS patients were younger (mean age: 64.7 ± 13.3 years,) compared to nUCSC patients (66.5 ± 12.0 years) or UCSC patients (67.0 ± 13.1 years), had the highest rate of an index TIA (36.6% vs. 25.8% in nUCSC & 28.8% in UCSC). Intravenous thrombolysis was more often administered in nUCSC patients (25.9%) if compared to patients treated in UCSC (20.6%) or PSC (19.9%, Table 1). Endovascular treatment was more often performed in nUCSC patients (4.1%) and UCSC patients (3.8%), if compared to patients treated in PSC (0.2%, Table 1. The median NIHSS score on admission was highest in nUCSC patients (3 points, interquartile range (IQR: 1-5, compared to UCSC and PCS patients (2 points, IQR 1–4 for UCSC and 2 points, IQR 1-4 for PCS; Table 1). The median duration of the in-hospital stay was 7 days (IQR 5-9) and differed at stroke unit level, as PCS patients stayed longer in-hospital (9 days (IQR 7,11)) if compared to UCSC patients (7 days (IQR 5-9)) and nUCSC patients (7 days (IQR 5-9)), respectively (Table 1).

**Table 1:** Baseline characteristics of 3431 MonDAFIS patients according to stroke unit level admission

	University-based comprehensive stroke centre (n = 1606)	Non univ.-based comprehensive stroke centre (n = 892)	Primary stroke centre (n = 933)	p-value
Age, years (mean, SD)	67.0 (13.1)	66.5 (12.0)	64.7 (13.3)	< 0.001
Female sex, n (%)	620 (38.6)	351 (39.3)	385 (41.3)	0.414
BMI, kg/m^2^ (mean (SD))	27.2 (5.1)	27.7 (4.9)	27.8 (4.9)	0.006
Index stroke				< 0.001
Transient ischaemic attack, n (%)	460 (28.8)	229 (25.8)	341 (36.6)	
Ischaemic stroke, n (%)	1140 (71.3)	659 (74.2)	590 (63.4)	
NIHSS score on admission, points (median [IQR])	2 [1,4]	3 [1,5]	2 [1,4]	< 0.001
Intravenous thrombolysis, n (%)	329 (20.6)	230 (25.9)	186 (19.9)	0.003
Endovascular treatment, n (%)	60 (3.8)	36 (4.1)	2 (0.2)	< 0.001
Hemicraniectomy, n (%)	2 (0.1)	2 (0.2)	-	0.287
Known cardiovascular risk factors on admission				
Diabetes mellitus, n (%)	423 (26.7)	220 (25.0)	239 (25.7)	0.629
Hypertension, n (%)	1,214 (76.8)	692 (78.5)	703 (75.6)	0.349
Congestive heart failure, n (%)	47 (3.0)	20 (2.3)	30 (3.2)	0.443
Hypercholesterolemia, n (%)	867 (54.8)	497 (56.5)	434 (46.7)	< 0.001
Coronary heart disease, n (%)	205 (13.0)	114 (12.9)	96 (10.3)	0.109
Peripheral arterial disease, n (%)	73 (4.6)	36 (4.1)	24 (2.6)	0.032
Renal impairment, n (%)	102 (6.5)	91 (10.3)	69 (7.4)	0.003
Sleep apnea, n (%)	53 (3.4)	30 (3.4)	13 (1.4)	0.005
Smoking/history of smoking, n (%)	809 (50.8)	464 (52.4)	400 (43.2)	< 0.001
Prior ischaemic stroke, n (%)	320 (20.2)	133 (15.1)	129 (13.9)	< 0.001
Prior TIA, n (%)	86 (5.4)	37 (4.2)	27 (2.9)	0.010
Prior intracranial bleed, n (%)	20 (1.2)	6 (0.7)	13 (1.4)	0.282
stroke unit beds, (median [IQR])	12 [12,20]	11 [6,18]	8 [8,10]	< 0.001
In-hospital stay, days (median [IQR])	6 [5,9]	7 [5,9]	9 [7,11]	< 0.001
Intervention group	799 (49.8)	446 (50.0)	469 (50.3)	0.970

Data are n (%), mean (SD), or median (IQR). *TIA* transient ischaemic attack. *NIHSS* National Institutes of Health Scale

P values stem from exact Fisher’s Tests, oneway ANOVA, or Kruskal–Wallis-test depending on the scaling of variables

### Stroke unit facilities

The median number of stroke unit beds in all participating stroke centres was 10 (IQR 6-12). UCSCs (12 beds (IQR 12-20)) and nUCSCs (11 beds (IQR 6-18)) had more stroke unit beds than PCSs (8 beds (IQR 8-10), Table 1). A structured analysis of monitor-based ECG recordings was reported to be performed at least once a day in 82.6% of all UCSC, 79.0% of all PCS and 72.0% of all nUCSC (Table 2). Automated AF detection was more frequently used in PSC (55.6%) compared to UCSC (27.3%) and nUCSC (27.4%). A higher rate of loop-recorder implantations in-hospital was reported in PSC patients (8.2%), if compared to nUCSC patients (1.1%; OR = 0.21, 95%CI 0.06–0.75) or UCSC patients (0.6%; OR = 0.14, 95%CI 0.04–0.51, Additional file 1: Table S2).

**Table 2:** In-hospital ECG-monitoring and cardiac imaging according to routine diagnostic care during the in-hospital stay after the index stroke by stroke unit levels

	University-based comprehensive stroke centre (n = 1606)	Non univ.-based comprehensive stroke centre (n = 892)	Primary stroke centre (n = 933)	p-value
ECG in hospital				
12-channel ECG, (median [IQR])	1 [1,1]	1 [1,1]	1 [1,1]	
0 ECG, 12-channel	42 (2.7)	5 (0.6)	19 (2.0)	< 0.001
1 ECG, 12-channel	1,469 (92.7)	781 (88.4)	799 (85.8)	
≥ 2 ECG, 12-channel	73 (4.6)	97 (11.0)	113 (12.1)	
24-h Holter-ECG, n (median [IQR])	0 [0,1]	1 [0,1]	1 [1,1]	
0 Holter-ECG, 24-h	838 (52.9)	257 (29.1)	152 (16.3)	< 0.001
1 Holter-ECG, 24-h	702 (44.3)	599 (67.9)	572 (61.4)	
≥ 2 Holter-ECG, 24-h	43 (2.7)	26 (2.9)	207 (22.2)	
Stroke Unit-Monitoring, h (median [IQR])	74 [48,89]	60 [28,74]	73 [45,90]	
≤ 24 h	64 (4.1)	89 (10.1)	26 (2.8)	< 0.001
> 24 to ≤ 72 h	617 (39.1)	525 (59.8)	400 (43.0)	
> 72 h	895 (56.8)	264 (30.1)	505 (54.2)	
Automated AF detection during stroke unit monitoring, n (%)	438 (27.3)	244 (27.4)	519 (55.6)	< 0.001
Daily rhythm visit @ stroke unit, n (%)	1,327 (82.6)	642 (72.0)	741 (79.4)	< 0.001
Echocardiography, n (%)	1,167 (74.0)	753 (85.4)	841 (90.3)	< 0.001
No echocardiography	411 (26.0)	129 (14.6)	90 (9.7)	
Transthoracic, n (%)	500 (31.1)	286 (32.1)	442 (47.4)	
Transesophageal ± transthoracic, n (%)	667 (42.3)	467 (52.9)	399 (42.9)	
Implanted devices during in-hospital stay				
Pacemaker, n (%)	2 (0.1)	1 (0.1)	3 (0.3)	0.569
Loop-Recorder, n (%)	10 (0.6)	10 (1.1)	76 (8.2)	< 0.001

Data are n (%), mean (SD), or median [IQR]. *H* hour

P values stem from exact Fisher’s Tests

### Duration of stroke unit stay

**Fig. 1:**
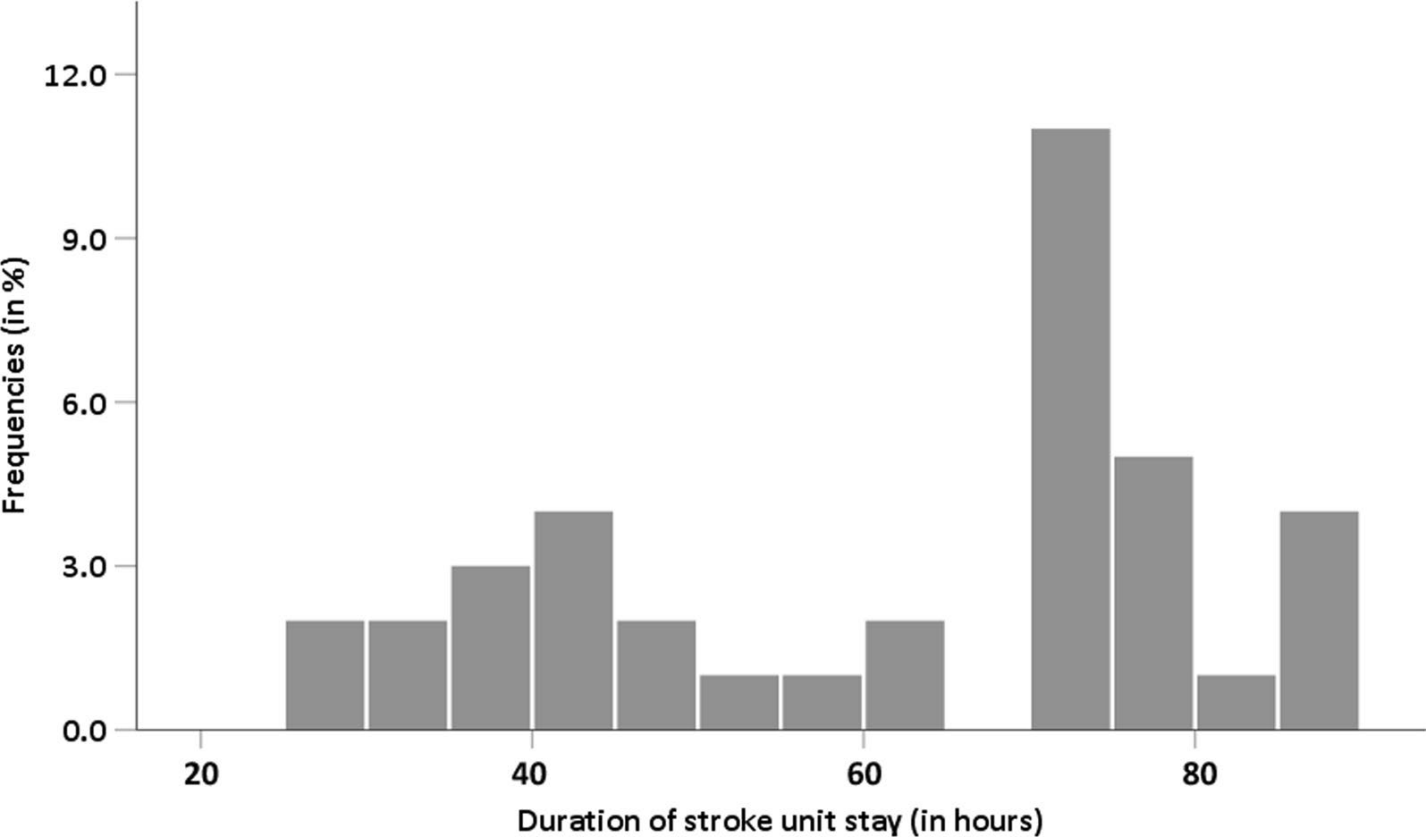
Histogramm of the durations of the stroke unit stay (in hours) of the index event across all stroke centres (n = 38)

The median duration of the stroke unit stay in 3431 study patients was 72 h (IQR 42–86). Duration of stroke unit stay was ≤ 24 h in 179 (5.3%) study patients, between > 24 and ≤ 72 h in 1542 (45.6%) patients, and > 72 h in 1664 (49.2%) patients. The distribution of the median duration of the stroke unit stay of all stroke centres is depicted in Fig. 1. Using a multivariable ordinal regression, patients with an index stroke (OR = 1.72, 95%CI 1.45–2.05 vs. TIA) and a more severe stroke on admission (OR = 1.60, 95%CI 1.30–1.96 vs. NIHSS score < 5 points) spend more time at the stroke unit (Table 3), while the duration of the stroke unit stay was not associated with sex and age. Median duration of the stroke unit stay differed at stroke unit level in the univariate analyses and was shorter in nUCSC patients (60 h, IQR 28–74) vs. UCSC patients (74 h, IQR 48–89) and PCS patients (73 h, IQR 45–90), respectively. Compared to UCSC and PCS, a stroke unit stay > 72 h was less frequently found in nUCSC patients (Fisher-exact test: p < 0.001, Table 2). However, multivariable ordinal regression showed a similar duration of the stroke unit stay at stroke unit level (Table 3).

**Table 3:** Multivariable mixed ordinal logistic regression model for the duration of stroke unit stay (≤ 24 h, > 24- ≤ 72 h, or > 72 h) in 3355 patients (random intercept models, random intercept for the 38 centres)

	Duration of stroke unit stay (≤ 24 h, > 24-72 h, > 72 h)		
	Adjusted OR	95% CI	P-value
Sex			
Male	1		
Female	0.88	0.75–1.03	0.112
Index event			
TIA	1		
Stroke	1.72	1.45–2.05	< 0.001
Stroke severity			
NIHSS < 5 points	1		
NIHSS ≥ 5 points	1.60	1.30–1.96	< 0.001
Age category			
≥ 75 years	1		
60–74 years	1.04	0.87–1.26	0.654
< 60 years	0.84	0.68–1.02	0.078
Type of stroke centre			
PCS	1		
UCSC	1.95	0.63–6.07	0.248
nUCSC	0.72	0.24–2.14	0.555

The regression model was additionally adjusted for duration of the in-hospital stay

*OR* odds ratio, *CI* confidence interval, *NIHSS* National Institutes of Health Scale, *TIA* transient ischaemic attack, *PCS* primary stroke centre, *UCSC* university-based comprehensive stroke centre, *nUCSC* non university-based comprehensive stroke centre

### Holter-ECG monitoring

**Fig. 2:**
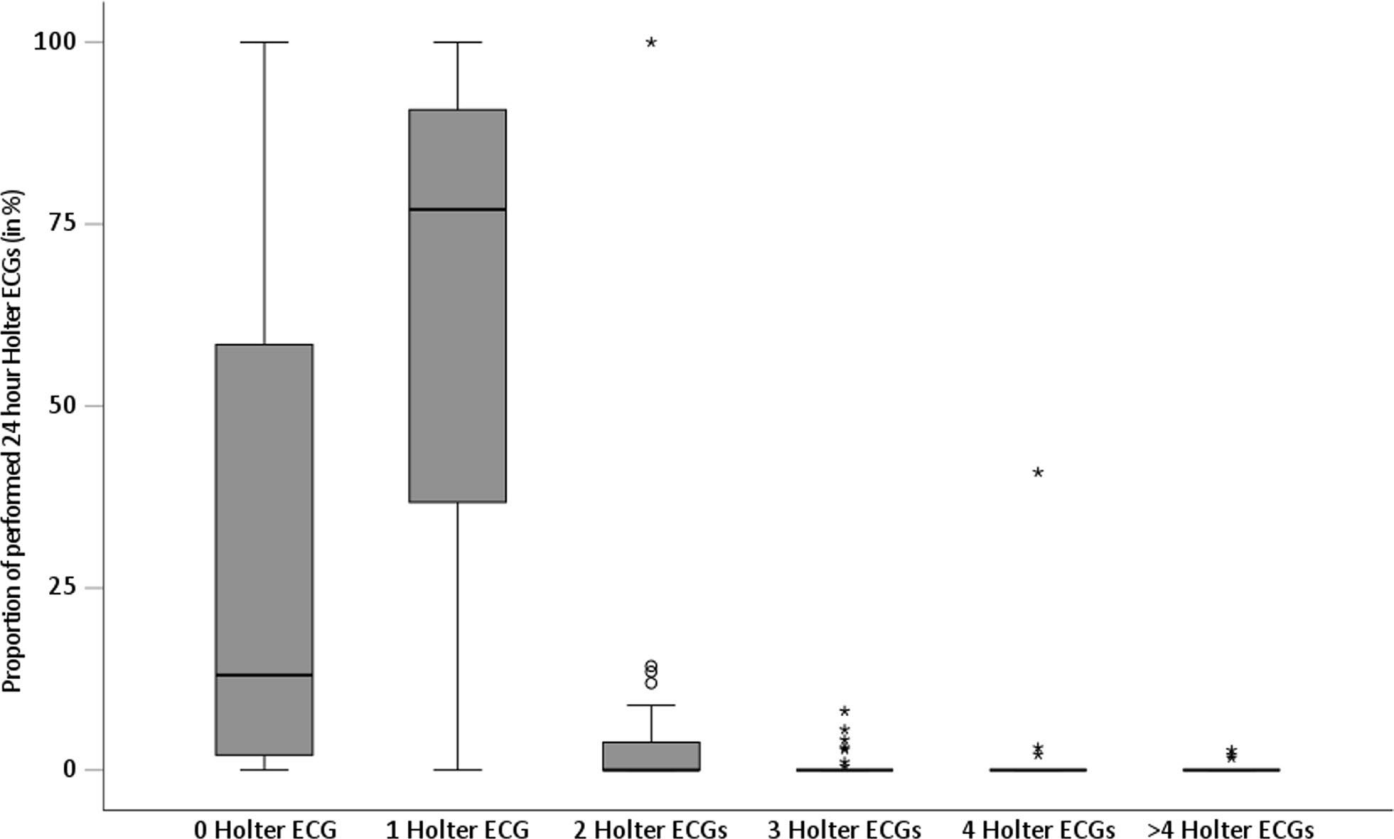
Bloxplots: relative frequencies of the number of performed 24-h Holter-ECGs during the in-hospital stay of the index event separately for the number of performed 24-h Holter-ECGs across all stroke centres (n = 38)

Overall, 2149 (63.3%) of 3396 patients received at least one 24-h Holter-ECG in-hospital in addition to stroke unit ECG monitoring, including 276 (8.1%) patients with two or more Holter-ECGs. The median number of 24-h Holter-ECGs per patient and centre was 2 (IQR 1–2). The distribution of 24-h Holter-ECG rate per patient in stroke centres is depicted in Fig. 2. Multinomial logistic regression analysis revealed no association of type of index stroke/TIA, stroke severity on admission or patients’ age with the use of 24-h Holter-ECGs (Additional file 1: Table S1). Female patients less frequently received two or more 24-h Holter-ECGs (vs. male: OR = 0.59, 95%CI 0.39–0.90). The median number of 24-h Holter-ECGs differed at stroke unit level, with a lower rate at UCSC (0, IQR 0-1) and nUCSC (1, IQR 0–1) compared to PCS (1, IQR 1-1), respectively (Table 2). Two or more 24-h Holter-ECGs were less often conducted in UCSC patients vs. PCS patients (OR = 0.03, 95%CI 0.00–0.64), while lower rates in nUCSC vs. PCS patients were less consistent (OR = 0.20, 95%CI 0.02–2.48).

### Echocardiography

**Fig. 3:**
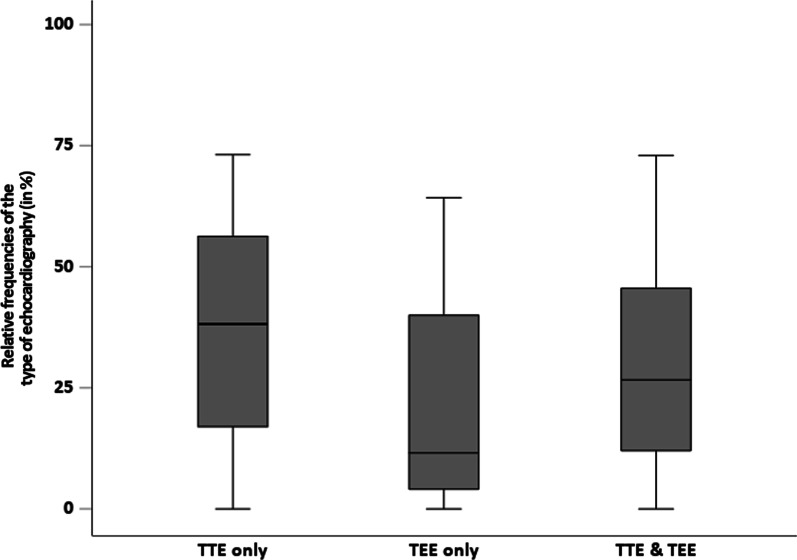
Boxplots: relative frequencies of the type of performed echocardiography during the in-hospital stay of the index event across all stroke centres (n = 38) stratified for transthoracic echocardiography (TTE) only, transesophageal echocardiography (TEE) only and the combination of TTE and TEE per centre. Wide interquartile ranges of all three categories demonstrate high variability in performing echocardiography across all stroke centres

In the MonDAFIS study, 1228 (36.2%) of 3391 study patients underwent TTE only, while 1533 (45.2%) patients underwent TEE (including 1020 patients with additional TTE). The median TEE rate per stroke centre was 54.3% (IQR 34–65), while the median TTE frequency rate per stroke centre was 77.0% (IQR 40–85). Multinomial analysis showed no substantial association of sex, type of index stroke, stroke severity on admission with the probability of TEE or TTE use (Table 4). Patients < 60 years of age had a higher likelihood to undergo TEE (OR = 3.44, 95%CI 2.67–4.42) compared to patients ≥ 60 years (Table 4). The distribution of TTE rates, TEE rates and the rate of TTE + TEE by stroke centres is depicted in Fig. 3. At stroke unit level, TEE (alone or in combination with TTE) was more frequently conducted in nUCSC patients (52.9%) compared to UCSC (42.3%) and PSC (42.9%), respectively (Fisher-exact test: p < 0.001, Table 2). Compared to PSC patients, UCSC patients less likely underwent TTE (OR = 0.21, 95%CI 0.05–0.85) or TEE (± TTE) (OR = 0.47, 95%CI 0.12–0.99, Table 4). Comparing nUCSC to PSC, TTE or TEE (± TTE) rates were similar.

**Table 4:** Multivariable multinomial mixed logistic regression model (random intercept model with random intercepts for the 38 centres) for the type of echocardiography using “no echocardiography” as reference group (n = 3361 patients)

	Transthroracic echocardiography			Transesophageal (± transthroracic) echocardiography		
	Adjusted OR	95% CI	P-value	Adjusted OR	95% CI	P-value
Sex						
Male	1			1		
Female	0.97	0.78–1.22	0.819	0.92	0.74–1.14	0.441
Index event						
TIA	1			1		
Stroke	1.16	0.90–1.48	0.254	1.14	0.89–1.45	0.303
Stroke severity						
NIHSS < 5 points	1			1		
NIHSS ≥ 5 points	1.05	0.78–1.40	0.757	1.02	0.77–1.34	0.916
Age category						
≥ 60 years	1			1		
< 60 years	0.84	0.64–1.11	0.226	3.44	2.67–4.42	< 0.001
Type of stroke centre						
PCS	1			1		
UCSC	0.21	0.05–0.85	0.028	0.34	0.12–0.99	0.047
nUCSC	0.73	0.19–2.82	0.650	0.78	0.27–2.26	0.652

The regression model was additionally adjusted for duration of the in-hospital stay and randomisation in MonDAFIS

*OR* odds ratio, *CI* confidence interval, *NIHSS* National Institutes of Health Scale, *TIA* transient ischaemic attack, *PCS* primary stroke centre, *UCSC* university-based comprehensive stroke centre, *nUCSC* non university-based comprehensive stroke centre

### In-hospital detection of a first episode of AF

AF was detected in 44 (2.8%) of 1580 UCSC patients, 28 (3.2%) of 882 nUCSC patients, and 50 (5.4%) of 931 PCS patients during the hospital stay of the index event. AF was less often detected in UCSC patients vs. PSC patients (OR = 0.65, 95%CI 0.45–0.93); while there was no difference in UCSC patients vs. PSC patients (OR = 0.69, 95%CI 0.46–1.04). Focussing on the duration of the stroke unit stay only, the AF detection rate did not differ substantially (Additional file 1: Table S3).

## Discussion

The results of our post-hoc analysis of the prospective, multi-centre MonDAFIS study deliver valuable insights into routine diagnostic cardiac work-up at German stroke units, including ECG monitoring and cardiac imaging.

The observed median duration of the stroke unit stay was 72 h (IQR 42-86), which is in line with ESC guideline recommendations for AF detection [7, 16]. In addition to stroke unit monitoring, 63% of all study patients underwent 24-h Holter-ECG during the in-hospital stay, including 8% with two or more 24-h Holter-ECGs. As recommended in a position paper of the German Heart and Brain Consortium [4] and the German stroke unit certification criteria [10, 11], a structured analysis of stroke unit monitoring data was reported by 26 of 38 stroke centres participating in the MonDAFIS study, enrolling 79% of all 3431 study patients in total. Interestingly, 14 of 38 stroke centres used an automated AF detection algorithm at the stroke unit during study conduct. AF detection rate according to routine care was 3.6% in all study patients, which is rather low if compared to previous cohort studies [4], and mainly based on the fact that MonDAFIS also included stroke patients aged less than 60 years (Haeusler AHJ 2016).

While German stroke unit certification requirements include a minimum TEE rate of 15% of all stroke patients treated [10, 11], a recent position paper of the European Stroke Organisation Executive Committee did not include a minimum TEE rate [17] and guideline recommendations are vague (Schnabel, Haeusler 2020). The median TEE rate across stroke centres participating in MonDAFIS was 54% and the IQR ranged from 34 to 65% across stroke centres. In comparison, the median TTE rate was 77.0% and IQR ranged from 40 to 85%, indicating the variability of echocardiography use across stroke centres, as similarly reported in the four study centres of the prospective FIND-AF_randomized_ trial [18]. Our data on TTE use are in line with a recent nationwide analysis using certification data of 310 German stroke units. However, median TEE rate in unselected patients with acute ischaemic stroke or TIA was reported to be 21.3% (IQR 16.4–29.5) in the nationwide analysis [13], indicating that TEE use in German stroke centres participating in prospective studies including stroke patients without AF at enrolment (like MonDAFIS or FIND-AF_randomized_) differs.

Patients younger than 60 years (vs. ≥ 60 years) underwent TEE (± TTE) three-times more likely, while there was no substantial association with patients´ age, type of index stroke or stroke severity. This is in line with recent recommendations for PFO closure in otherwise cryptogenic stroke patients up to 60 years [2].

Comparing hospitals with a certified regional (PSC) or comprehensive stroke unit in consideration of the university status comprehensive stroke units (nUCSC/UCSC), revealed that PCS patients compared to UCSC patients were more likely to receive a 24-h Holter-ECG, more often underwent echocardiography (TTE or TEE ± TTE), or loop recorder implantation. Comparing PCS patients to nUCSC patients, loop recorder were more frequently implanted. The duration of the stroke unit stay was similar at stroke unit level.

Interestingly, a first episode of AF was more often documented in PSC patients compared to UCSC patients, while there was no substantial difference when PSC patients were compared to nUCSC patients. By comparing routine cardiac work-up at stroke unit level, our result support the finding that AF detection rates in stroke patients increase with prolonged monitoring duration [15, 16], as PCS patients more often received additional 24-h Holter-ECGs.

One major strength of our post-hoc analysis of a large prospective study is the precise assessment of ECG monitoring and type of echocardiography. However, there are also limitations which have to be considered. First, the MonDAFIS study randomised non-AF patients only. Therefore, our findings cannot be transferred to cardiac work-up in hospitalized stroke patients with known AF (Herm et al., 2013) or to unselected cohorts of stroke patients [13]. Second, there is a selection bias on patient level, as informed consent was an inclusion criteria, excluding patients with dementia and very severe stroke. In addition, patients with severe stroke that might have died before cardiac work-up was started, which might introduce a potential bias for this study. However, only six out of 3431 MonDAFIS patients died in hospital after the index event. Furthermore, we cannot exclude a selection bias, as female study patients were underrepresented (39.5%), which may limit the generalizability of our findings. However, the gender distribution in MonDAFIS is in line with a recent meta-analysis of stroke trials [1]. Third, the open-label design of the MonDAFIS study may had an impact on the extent of routine diagnostic cardiac work-up despite otherwise recommended. However, we have controlled for this potential bias by including randomisation group as a covariate in all multivariable analyses. Fourth, AF diagnosis according to routine care was not externally validated, thus, false positive diagnosis of AF cannot be excluded. Fifth, only stroke units were included in the MonDAFIS study that fulfilled the certification criteria of the German stroke society. In addition, the 38 hospitals actively participating in the randomised MonDAFIS study do not necessarily represent the more than 340 certified stroke units in Germany. Finally, our results cannot be generalized to stroke centres world-wide, as hospital facilities and certification requirements differ outside of Germany.

## Conclusion

Routine diagnostic cardiac work-up during the in-hospital stay after acute ischaemic stroke or TIA varied to some extent in stroke centres participating in the MonDAFIS study with regard the use of echocardiography or 24-h Holter-ECG recording. The observed heterogeneity may be attributed to rather vaguely defined indication-criteria for cardiac diagnostic procedures in patients with acute ischaemic stroke or TIA and might be related to limited resources in stroke centres. Taken together, further efforts are needed to define the optimal extent of routine diagnostic cardiac work-up allocated to patients in the acute phase of stroke to optimize secondary stroke prevention.

## Supplementary Information

**Additional file 1: Table S1.** Multivariable mixed multinomial logistic regression model (random intercept model with random intercepts for the 38 centres) for the number of 24-hour-Holter ECG using “no 24-h Holter-ECG” as reference group (n= 3361 patients). The regression model was additionally adjusted for duration of the in-hospital stay and randomisation in MonDAFIS.** Table S2.** Multivariable mixed binary logistic regression model (random intercept model with random intercepts for the 38 centres) for the implantation of a loop recorder (yes/no) during the hospital stay of the index stroke in 3364 patients. The regression model was adjusted for duration of the in-hospital stay and randomisation in MonDAFIS.** Table S3.** Multivariable mixed binary logistic regression model (random intercept model with random intercepts for the 38 centres) for the detection of AF during the in-hospital stay of the index event and for the AF-detection during Stroke Unit-Monitoring of during the routine care according to stroke unit level*. The regression model was adjusted for sex, type of index event, stroke severity, age, and randomisation group.

## Data Availability

Deidentified participant data with corresponding data dictionary of the data underlying the current manuscript will be made available upon reasonable request to the corresponding author Prof. Matthias Endres (matthias.endres@chairte.de). Data will be shared to external researchers for scientific non-commercial purposes after approval of the proposal by the MonDAFIS steering board including a signed data access agreement.

## Abbreviations

AF: Atrial fibrillation
CI: Confidence interval
ESO: European Stroke Organisation
IQR: Interquartile range
MonDAFIS: Impact of standardized MONitoring for Detection of Atrial Fibrillation in Ischaemic Stroke
NIHSS: National Institutes of Health Stroke Scale
nUCSC: Non university-based comprehensive stroke centres
OR: Odds ratio
PCS: Primary stroke centres
SD: Standard deviation
TEE: Transesophageal echocardiography
TIA: Transient ischaemic attack
TTE: Transthoracic echocardiography
UCSC: University-based comprehensive stroke centre
