# Supplementary material for: Extent of routine diagnostic cardiac work-up at certified German stroke units participating in the prospective MonDAFIS study
Source: Neurol Res Pract. 2023 Jun 1;5:21. doi: 10.1186/s42466-023-00246-8 (PMC10233882; doi:10.1186/s42466-023-00246-8)
Supplement: Supplementary file 1 — Additional file 1: Table S1. Multivariable mixed multinomial logistic regression model (random intercept model with random intercepts for the 38 centres) for the number of 24-hour-Holter ECG using “no 24-h Holter-ECG” as reference group (n= 3361 patients). The regression model was additionally adjusted for duration of the in-hospital stay and randomisation in MonDAFIS. Table S2. Multivariable mixed binary logistic regression model (random intercept model with random intercepts for the 38 centres) for the implantation of a loop recorder (yes/no) during the hospital stay of the index stroke in 3364 patients. The regression model was adjusted for duration of the in-hospital stay and randomisation in MonDAFIS. Table S3. Multivariable mixed binary logistic regression model (random intercept model with random intercepts for the 38 centres) for the detection of AF during the in-hospital stay of the index event and for the AF-detection during Stroke Unit-Monitoring of during the routine care according to stroke unit level*. The regression model was adjusted for sex, type of index event, stroke severity, age, and randomisation group. [file 42466_2023_246_MOESM1_ESM.docx]

**Additional file 1**

**Table S1.** Multivariable mixed multinomial logistic regression model (random intercept model with random intercepts for the 38 centres) for the number of 24-hour-Holter ECG using “no 24-h Holter-ECG” as reference group (n= 3,361 patients). The regression model was additionally adjusted for duration of the in-hospital stay and randomisation in MonDAFIS.

|  | **One 24-hour-Holter ECG** | | | | **Two or more 24-hour-Holter ECGs** | | |
| --- | --- | --- | --- | --- | --- | --- | --- |
|  | **Adjusted OR** | **95% CI** | **P-value** | **Adjusted OR** | | **95% CI** | **P-value** |
| **Sex** |  |  |  |  | |  |  |
| male | 1 |  |  | 1 | |  |  |
| female | 1.23 | 0.98-1.54 | 0.070 | 0.59 | | 0.39-0.90 | 0.013 |
| **Index event** |  |  |  |  | |  |  |
| TIA | 1 |  |  | 1 | |  |  |
| stroke | 1.07 | 0.83-1.38 | 0.610 | 0.86 | | 0.56-1.33 | 0.507 |
| **Stroke severity on admission** |  |  |  |  | |  |  |
| NIHSS <5 points | 1 |  |  | 1 | |  |  |
| NIHSS ≥5 points | 0.80 | 0.61-1.04 | 0.099 | 0.63 | | 0.37-1.07 | 0.087 |
| **Age category** |  |  |  |  | |  |  |
| ≥ 60 years | 1 |  |  | 1 | |  |  |
| <60 years | 0.99 | 0.78-1.26 | 0.953 | 0.94 | | 0.61-1.45 | 0.787 |
| **Type of stroke centre** |  |  |  |  | |  |  |
| PCS | 1 |  |  | 1 | |  |  |
| UCSC | 0.13 | 0.02-1.09 | 0.060 | 0.03 | | 0.00-0.64 | 0.024 |
| nUCSC | 0.17 | 0.02-1.21 | 0.077 | 0.20 | | 0.02-2.48 | 0.207 |

OR=odds ratio, CI=confidence interval. NIHSS=National Institutes of Health Scale. TIA=transient ischaemic attack. PCS=primary stroke centre, UCSC=university-based comprehensive stroke centre, nUCSC non university-based comprehensive stroke centre.

**Table S2.** Multivariable mixed binary logistic regression model (random intercept model with random intercepts for the 38 centres) for the implantation of a loop recorder (yes/no) during the hospital stay of the index stroke in 3,364 patients. The regression model was adjusted for duration of the in-hospital stay and randomisation in MonDAFIS.

|  | **Implantation of a loop recorder** **during in-hospital stay** | | |
| --- | --- | --- | --- |
|  | **Adjusted OR** | **95% CI** | **P-value** |
| **Sex** |  |  |  |
| male | 1 |  |  |
| female | 0. 99 | 0. 63-1.56 | 0.970 |
| **Index event** |  |  |  |
| TIA | 1 |  |  |
| stroke | 1.30 | 0.79-2.14 | 0. 311 |
| **Stroke severity on admission** |  |  |  |
| NIHSS <5 points | 1 |  |  |
| NIHSS ≥5 points | 0.93 | 0.54-1.61 | 0.807 |
| **Age category** |  |  |  |
| ≥ 75 years | 1 |  |  |
| 60-74 years | 0.66 | 0. 41-1.07 | 0.090 |
| <60 years | 0.38 | 0.21-0.71 | 0.002 |
| **Type of stroke centre** |  |  |  |
| PCS | 1 |  |  |
| UCSC | 0.14 | 0.04-0.51 | 0.003 |
| nUCSC | 0.21 | 0.06-0.75 | 0.017 |

OR=odds ratio, CI=confidence interval. NIHSS=National Institutes of Health Scale. TIA=transient ischaemic attack. PCS=primary stroke centre, UCSC=university-based comprehensive stroke centre, nUCSC non university-based comprehensive stroke centre.

**Table S3:** Multivariable mixed binary logistic regression model (random intercept model with random intercepts for the 38 centres) for the detection of AF during the in-hospital stay of the index event and for the AF-detection during Stroke Unit-Monitoring of during the routine care according to stroke unit level*****. The regression model was adjusted for sex, type of index event, stroke severity, age, and randomisation group.

|  | **Detection of AF**  **during Stroke Unit-Monitoring** | | | **Detection of AF**  **according to routine care** | | |
| --- | --- | --- | --- | --- | --- | --- |
|  | n (%) | Adjusted OR [95%CI] | p-value | n (%) | Adjusted OR [95%CI] | p-value |
| **PCS** |  | 1 |  |  | 1 |  |
| **UCSC** | UCSC:  38 (2.4) vs.  PCS:  36 (3.9) | 0.81 [0.55-1.20] | 0.297 | UCSC:  44 (2.8) vs.  PCS:  50 (5.4) | 0.65 [0.45-0.93] | 0.017 |
| **nUCSC** | nUCSC:  12 (1.4) vs.  PCS:  36 (3.9) | 0.67 [0.42-1.06] | 0.086 | nUCSC:  28 (3.2) vs.  PCS:  50 (5.4) | 0.69 [0.46-1.04] | 0.077 |

OR=odds ratio, CI=confidence interval. NIHSS=National Institutes of Health Scale.*Detection of AF “according to routine care” encompasses AF-detection during Stroke Unit-monitoring and apart from Stroke Unit-monitoring. PCS=primary stroke centre, UCSC=university-based comprehensive stroke centre, nUCSC non university-based comprehensive stroke centre.
